# Molecular Electric Transducers as Motion Sensors: A Review

**DOI:** 10.3390/s130404581

**Published:** 2013-04-08

**Authors:** Hai Huang, Vadim Agafonov, Hongyu Yu

**Affiliations:** 1 School of Electrical, Computer and Energy Engineering, Arizona State University, Tempe, AZ 85287, USA; E-Mail: hhuang60@asu.edu; 2 Center of Molecular Electronics, Moscow Institute of Physics and Technology, Moscow 141700, Russia; E-Mail: agvadim@yandex.ru; 3 School of Earth and Space Exploration, Arizona State University, Tempe, AZ 85287, USA

**Keywords:** molecular electronic transducer, accelerometers, seismometers

## Abstract

This article reviews the development of a new category of motion sensors including linear and angular accelerometers and seismometers based on molecular electronic transducer (MET) technology. This technology utilizes a liquid not only as an inertial mass, but also as one of the main elements in the conversion of mechanical motion into electric current. The amplification process is similar to that in a vacuum triode. Therefore, it is possible to achieve signal amplification close to 10^8^. Motion sensors demonstrating wide frequency and dynamic range and sensitivity that are one to two orders of magnitude better than MEMS devices of the same size have been developed.

## 1. Introduction

Motion sensors, including accelerometers and gyroscopes, provide measurement of movement in at least six degrees of freedom. The simplest way to do motion sensing is with a solid-state mass-spring system, creating a damped simple harmonic oscillator. The movement of the solid-state proof mass can be measured with respect to displacement, velocity, or acceleration by suitable mechanical-electrical transducers. Efforts to miniaturize linear accelerometers and gyroscopes for inertial systems are mostly concentrated around Micro-Electro-Mechanical Systems (MEMS) technology. Similarly, in terms of design, fabrication, and readout, accelerometers and gyroscopes are the current leaders in commercially successful MEMS technology. Among a variety of transduction mechanisms underlying solid-state MEMS motion sensors, the most successful types are based on capacitive transduction due to the simplicity of the sensor element itself, no need for exotic materials, low power consumption, and good stability with respect to temperature. Although capacitive transducers have a characteristic nonlinear capacitance *vs.* displacement response, feedback is commonly used to convert the signal to a linear output. MEMS motion sensors in combination with other sensors, such as compass, pressure sensor, and GPS, have created a consumer electronics sensing package that works as the intelligent interface for users to interact with their electronics and, further on, with external environments, and have reasonable performance with low cost CMOS-compatible silicon microfabrication technology. However, in addition to high-volume consumer electronics markets requiring low-to-medium performance motion sensors, there are huge markets for high-performance motion sensing devices, with applications designed for military inertial navigation/guidance, high-resolution seismic sensing and high-g sensors. The key requirements for these high-performance applications include small size, wide bandwidth, low noise floor, low cross-axis sensitivity, low drift, wide dynamic range, high shock survivability, and low power consumption. There has been plenty of work done along the direction of scaling down the device size while maintaining low noise, high sensitivity and high resolution using MEMS techniques [1–3]. However, the design and fabrication of these solid-state MEMS devices are complicated, which can result in low reliability, low reproducibility, and high cost. More importantly, they have yet to prove satisfactory for specific applications, especially in low frequency seismic sensing, because of the inherent limitation of the working mechanism of the solid-state mass-spring system. For some applications they also have limited successes due to their fragility to high shocks.

As opposed to a solid inertial mass, a molecular electronic transducer (MET) is sensitive to the movement of a liquid electrolyte relative to fixed electrodes. METs are part of a third class of fundamental electronic devices, characterized by charge transfer via ions in solution—hence the name “Solion”. This is in contrast to solid-state electronics (charge transfer by electron/hole pairs in a solid conductor or semiconductor) and vacuum tubes (charge transfer by free electrons in an ionized gas or vaccum). Solion technology was first developed in the 1950s by US-Navy sponsored research. Early applications of Solion devices were for detection of low-frequency acoustic waves, either in the form of an infrasonic microphone or limited-band seismometer [4–7]. Significant work on Solion motion detectors was continued in Russia, where the term “Molecular Electronic Transducer” was introduced to describe such a device [8]. Inspired by the exceptionally high rate of mechanical signal conversion to electric current in MET involving mass and charge transport, pioneering MET studies [9–15] provide an alternative paradigm in the development of motion sensors in wide variety of applications including nuclear explosion monitoring and seismic sensing in planetary exploration [16–18]. The advantages of MET motion sensors include their small size, lack of fragile moving parts (thus high shock tolerance), high sensitivity and low noise especially at low frequencies, and independence of the response on installation angle.

## 2. Molecular Electronic Transducer-Principle of Operation

**Figure 1.**
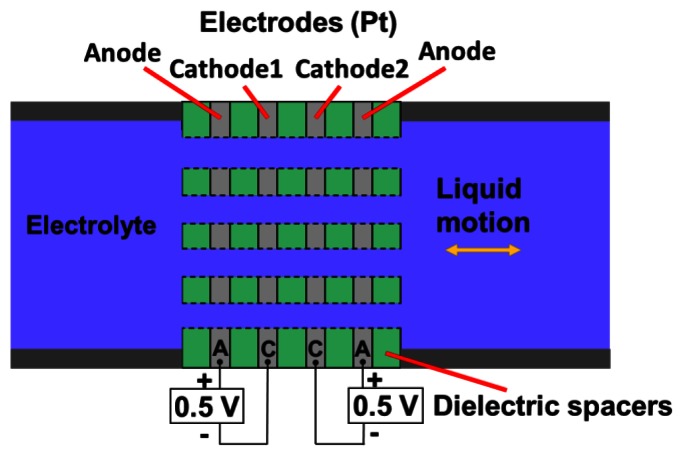
Schematic of the basic MET sensing element.

Figure 1 illustrates the basic concept of a MET sensing element, consisting of an electrochemical cell with two anode-cathode pairs. An electrolyte-filled channel allows the electrolyte to move inertially along the channel's length. Four electrodes configured as anode-cathode-cathode-anode (ACCA) separated by dielectric layers span the width of this channel. Holes through the electrodes allow the flow of electrolyte. Each anode-cathode pair is an electrochemical cell, in which charges are transferred between anode and cathode by ions in the electrolyte. Traditionally, standard machined platinum (Pt) mesh is used to make the electrodes, and plastic or ceramic grids produce the dielectric inter-electrode spacers [19]. This approach is convenient to quickly produce samples, but significantly limits the possible range of the geometrical parameters of the cell and consequently the optimization possibilities of the sensing cell. Meanwhile making sizes of elements of the cell smaller expands the frequency range and decreases convection produced noise inside the sensitive cell. An alternative approach to build the sensing element is to use (MEMS) techniques, which have reduced the internal dimensions of MET electrodes close to 1 μm, improved the sensitivity and reproducibility [18].

The sensing mechanism of the MET is based on using of the electrochemical cell where reversible chemical reactions transfer charge between anode and cathode via electrolyte ions in solution. Typically, MET uses concentrated iodine-iodide electrolyte containing potassium iodide (KI) or lithium iodide (L_i_I) and a small amount of elemental Iodine (I_2_) [20]. In the presence of iodide, iodine turns into a soluble compound, tri-iodide, as follows [20]:

(1) $$I_{2}+I^{-}\to I_{3}^{-}$$

when the electrodes are biased and thus current passes through the electrochemical cell, the following reversible active electrochemical reactions occur on the electrodes:

On cathodes, reduction of tri-iodide:

(2) $$I_{3}^{-}+2e^{-}\to 3I^{-}$$

The reverse reaction takes place on anodes:

(3) $$3I^{-}-2e^{-}\to I_{3}^{-}$$

The electrical current through the solid/liquid interface becomes possible because of the tri-iodide ions presence in the solution. That's why this component of the solution is called active. According to Equation (2), the interface charge transfer is associated with generation and absorption of the tri-iodide ions on the electrode surface. So the electrical current through any electrode can be related to the flux of active ions toward or backward of the electrode according to the following:

(4) $$I=Dq(\oint_{S}\left(\nabla c,n\right)dS$$

where *D* is the diffusion coefficient, *c* is the concentration of the active charge carriers, *q* is the charge transferred across the interface in single electrochemical reaction (two times absolute values of the electron charge in our case), ***n*** is a unit vector normal to the surface of the electrode, integration is done over *S*, electrode surface area. Here only diffusion is considered as mechanism responsible for the active ions transport in the electrolyte volume. The migration is not included due to the screening of the electrical field in the highly concentrated electrolyte and the convection does not contribute to charge transfer through the electrode surface due to zero-velocity condition on the solid surface.

The operation principle of MET can be described as follows: when electric voltage is applied to the system, electrochemical current (background current) appears, regardless of the presence of mechanical motion of the electrolyte. As the inter-electrode voltage is increased, the reaction rates on the electrodes increase too. Finally, in the situation when any tri-iodide ion arrives the cathode immediately participates in the electrochemical reaction in Equation (2), further increase of the voltage does not change the current and the saturation regime occurs. In this regime the cathode current is sensitive to variation of volumetric transport of tri-iodide ions. Anode current variations follow the cathode ones, keeping the electrolyte uncharged. In the presence of a mechanical motion input, electrolyte starts moving due to inertia, and convective transport of ions changes the electrode current according to the mechanism described above. The difference of the cathode currents in two anode-cathode pairs is employed as the output signal for a MET motion sensor. Although each electrode current is non-linear with respect to fluid velocity, the combined output of both cathodes is linear for a very wide range of fluid velocities. Mathematically, the sensor's output current is given by [20]:

(5) $$I_{\text{out}}=I_{C2}-I_{C1}=\;Dq(\oint_{S_{C2}}\left(\nabla c,n\right)dS_{C2}-\oint_{S_{C1}}\left(\nabla c,n\right)dS_{C1}$$

where *I*_C1_, *I*_C2_ are the currents through the surface of the corresponding cathodes, S_C1_, S_C2_ are the surface areas of the corresponding cathodes.

### 2.1. Transfer Function

A signal conversion in MET motion sensor can be considered as a superposition of two processes: first, input motion is converted to fluid motion of the electrolyte by mechanical system. Next, the electrolyte's velocity is measured by the electrochemical system, resulting in an output current of the sensor. Therefore, the frequency-dependent transfer function of the entire device can be written as:

(6) $$H\left(\omega \right)=\;H_{\text{mech}}(\omega )H_{ec}(\omega )$$

where *H_mech_*(*w*) describes the mechanical response of the fluidic system, which in the case of linear MET sensor, is analogous to a solid-state damped, driven harmonic oscillator. In this sense, the restoring force to the liquid inertial mass is provided by the rubber membranes which are located at the two ends of the channel to seal the electrolyte, and the damping force is caused by hydrodynamic resistance of the electrolyte as it flows through the sensing element. The equation that governs motion of the electrolyte can therefore be expressed as:

(7) $$\frac{d^{2}V}{dt^{2}}+\frac{R_{h}S_{ch}}{\rho L}\frac{dV}{dt}+\frac{k}{\rho L}V=-S_{ch}a$$

where *V* is the volume of fluid passing through the channel, *a* is the external acceleration, *R_h_* is the hydrodynamic resistance which is solely determined by the channel geometry in laminar flow condition, *k* is the coefficient of volume stiffness and depends only on the characteristics of the membrane, *ρ* is the density of the electrolyte, *S_ch_* is the cross-section area of the channel and *L* represents the length of the channel, filled with electrolyte. By transforming Equation (7) to the frequency domain, the magnitude of the transfer function of the fluid mechanical motion in frequency domain can be obtained as follows:

(8) $$\left|H_{\text{mech}}\left(\omega \right)\right|=\left|\frac{Q(\omega )}{a(\omega )}\right|=\frac{\rho L}{\sqrt{{\left(\frac{\rho L}{S_{ch}}\right)}^{2}\frac{{\left(\omega^{2}-\omega_{0}^{2}\right)}^{2}}{\omega^{2}}+R_{h}^{2}}}$$

where $Q(t)=\frac{dV(t)}{dt}$ is the volumetric flow rate.

*H_ec_*(*ω*) in Equation (4) describes the ability of the electrochemical system to detect electrolyte motion as a function of frequency. A simple model for *H_ec_*(*ω*) was derived analytically by Larcam [7], having a form of:

(9) $$\left|H_{ec}\left(\omega \right)\right|=\left|\frac{I(\omega )}{Q(\omega )}\right|=\;\frac{C}{\sqrt{1+{\left(\frac{\omega }{\omega_{D}}\right)}^{2}}}$$

where *C* (*A/(m^3^/s)*) is the conversion factor of the electrochemical cell, *ω_d_* = D/d^2^ is the diffusion frequency and *d* is the inter-electrode distance. In-depth characterization of the above electrochemical subsystem is based on the analytical and numerical solution of the following partial differential equations [11,15,21–24]:

(10.1)(10.2)(10.3) $$\{\begin{array}{c} \rho \left(\frac{\partial \text{u}}{\partial t}+\left(\text{u}\cdot \nabla \right)\text{u}\right)=-\nabla P+\mu \nabla^{2}\text{u}\;\\ \nabla \cdot \text{u}=0\;\\ \frac{\partial c}{\partial t}+\nabla \cdot \left(-D\nabla c+c\text{u}\right)=0\; \end{array}$$

Equation (10.1) is the time domain Navier-Stokes equation describing momentum balances, where *ρ* denotes the density (kg/m^3^),**u** is the velocity (*m/s*), *μ* denotes dynamic viscosity (*P*α·s), and P equals pressure (*P*α). Equation (10.2) is the continuity equation for incompressible flow. The boundary conditions include a known pressure difference which drives the flow through the channel and the velocity is zero at the wall. Equation (10.3) is the diffusion-convection Nernst-Planck equation showing the tri-iodide transport balance. Usually, on cathodes, zero concentration condition is used, which corresponds to the saturation current regime described above. Dielectric surfaces are considered as non-penetrable for the ions (zero flow condition). For anodes, fixed concentration condition is frequently used, although this sort of condition can't be considered as well-founded. The problem of the correct formulation of the boundary condition on anode is discussed in [24]. Fortunately, the boundary condition on anodes has little effect on cathodes currents difference which is used as the system output signal.

Several theoretical analyses have been conducted on the electrochemical system frequency response at both low and high frequencies [11,21,22]. At low frequencies, where diffusion length $\lambda_{d}=\sqrt{D/\omega }$ appears to be much higher than the characteristic dimension which is the inter-electrode spacing *d* of the four-electrode structure:

(11) $$I\overset{\omega \to 0}{\to }\text{Q}\cdot \text{const}$$

Therefore, using Equation (8):

(12) $$H\left(\omega \right)\overset{\omega \to 0}{\to }\text{const}\cdot \omega$$

As frequency increases, the sensitivity starts to decay as *λ_d_* becomes lower than the characteristic dimension of the electrode system *d*. For the mesh electrodes made with cylindrical wires, and *ω* ≫ *D*/*d*^2^, the amplitude of cathode current yields:

(13) $$I\overset{\omega \gg D/d^{2}}{\to }Q\cdot \omega^{-3/2}$$

Therefore, using Equation (8):

(14) $$H\left(\omega \right)\overset{\omega \gg D/d^{2}}{\to }\text{const}\cdot \omega^{-5/2}$$

### 2.2. Feedback Subsystem

**Figure 2.**
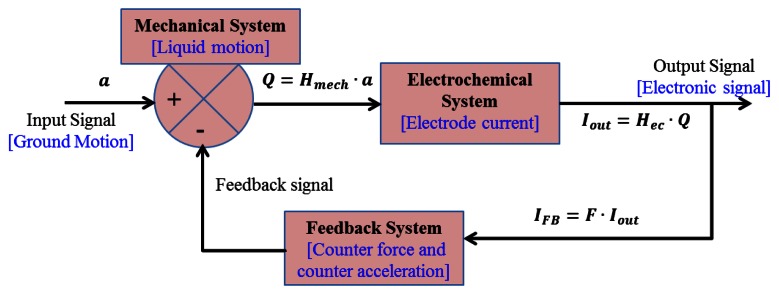
A block diagram of MET motion sensor including feedback subsystem.

The feedback subsystem is added to allow an additional controllable force to act on the mechanical system. The feedback system herein introduces a layer of complexity into the overall system's transfer function. The simplified block diagram shown as Figure 2 illustrates how the input signal is manipulated by each subsystem. Text in blue describes the physical parameters being affected or measured in each step. The input signal is processed by the mechanical and electrochemical systems in order to produce the output signal, which is in turn used to adjust the mechanical system by the feedback system. The feedback current I_FB_ is simply the output current modified by a feedback parameter F. Finally, the effect of the feedback is applying to the inertial mass with additional counterforce, opposite to inertial force, and consequently effectively decreasing the input acceleration. In the feedback system described above, a higher output signal gives negative feedback impeding fluid flow, thereby increasing the dynamic range of measurable signals. Moreover, this could be used to adjust sensitivity frequency response, allowing desired signals in certain bandwidth.

### 2.3. Noise

The main contributors to the self-noise of the MET sensor are the thermohydrodynamic self-noise, convection-induced self-noise, and geometry noise [10,25].

#### 2.3.1. Thermohydrodynamic Self-Noise

The thermohydrodynamic self-noise is generated from the fluctuations of the pressure difference on both sides of the channel of MET sensor. In units of input acceleration, this noise spectral density is frequency independent:

(15) $${{\langle a\rangle }^{2}}_{\omega }=\frac{2k_{B}\cdot T\cdot R_{h}}{\rho^{2}l^{2}}$$

where *T* is the absolute temperature, *k_B_* is Boltzmann's constant, *ρ* is the electrolyte density, *l* is the length of the transducer channel, *R_h_* is the hydrodynamic impedance of the transducer which is given by:

(16) $$R_{h}=\frac{8\mu l}{\pi r^{4}}$$

where *r* is the radius of the circular cross-section of the channel and *μ* is the viscosity of the fluid. It can be seen that the lower *R_h_*, higher *ρ* and *l* result in lower thermohydrodynamic self-noise. For a typical MET sensor with *R_h_* = 5×10^8^(N·S)/m^5^ and *l* = 5×10^-2^, the theoretical thermohydrodynamic self-noise is $\;3ng/\sqrt{Hz}$.

#### 2.3.2. Convection-Induced Self-Noise

The convection-induced self-noise is the result of the natural convection of the liquid [25]. Even if the liquid as a whole is stable, small local variations of the liquid density produce flows in the liquid. These flows generate an additional noise in the MET cell output current. This process is difficult to describe analytically. However, numerical modeling and experimental results can provide enough data to calculate the convection self-noise and can be used to predict its value for any specific configuration of the MET cell. In general, the value of the convection induced self-noise depends on the Rayleigh number (Ra) of the electrolyte. It has been proven both experimentally and numerically that the convection self-noise decreases as Ra decreases. Lower Ra corresponds to a lower electrolyte concentration and smaller typical dimensions of the elements of the transducer cell. On the other hand, Ra cannot be too small because that would correspond to a very small size for the transducer cell and very small concentration of the electrolyte, which usually means high hydrodynamic impedance (high thermohydrodynamic noise) and low sensitivity of the transducer, respectively.

#### 2.3.3. Geometry Self-Noise

The geometry self-noise is proportional to the electrochemical part of the transfer function of MET transducer. To calculate the geometry self-noise, the following equation can be used:

(17) $${{\langle v\rangle }^{2}}_{f}=\beta \frac{4k_{B}T}{R_{h}}{\bar{k_{f}}}^{2}\alpha^{2}$$

where $\bar{k_{f}}$ is the averaged electrochemical part of the transfer function, *α* is the transducer conversion coefficient from output noise current to input noise velocities, and *β* is an empirical coefficient. The geometry noise is the dominant one at very low frequency.

#### 2.3.4. Shot Noise

The shot noise of the MET sensor is given by the following formula:

(18) $${\langle v^{2}\rangle }_{f}=\frac{2qI}{K}$$

where *I* is the quiescent current passing through the cell, *K* is the transducer conversion coefficient, *q* is the absolute value of the charge passing through the electrode boundary in the elementary chemical reaction on the electrode. In the electrochemical system usually used in MET transducer, *q* equals 2*e*, where *e* is the absolute value of the charge of the electron.

#### 2.3.5. Electronic Self-Noise

Electronic self-noise of MET sensor comes from the signal conditioning electronics, including the current to voltage converter and filters. The filters have unity gain in the proposed instrument pass band and do not contribute to the electronic self-noise. The equivalent input-referred electronic noise can be written as follows:

(19) $${\langle v^{2}\rangle }_{f}=\frac{I_{f}^{2}+U_{f}^{2}(\frac{1}{R^{2}}+\frac{1}{{\left|Z\right|}^{2}})}{K^{2}}$$

where *R* is the resistor in the feedback of the operational amplifier used to convert the current into voltage, *Z* is the impedance of the MET cell, $I_{f}^{2}$ and $U_{f}^{2}$ are voltage and current noise spectrum density of the operational amplifier, respectively. To decrease the electronic self-noise, the transducer should have a high impedance and conversion factor. Also, low-noise operational amplifiers should be used. In all MET devices, the electronic self-noise contributes at relatively high frequencies.

**Figure 3.**
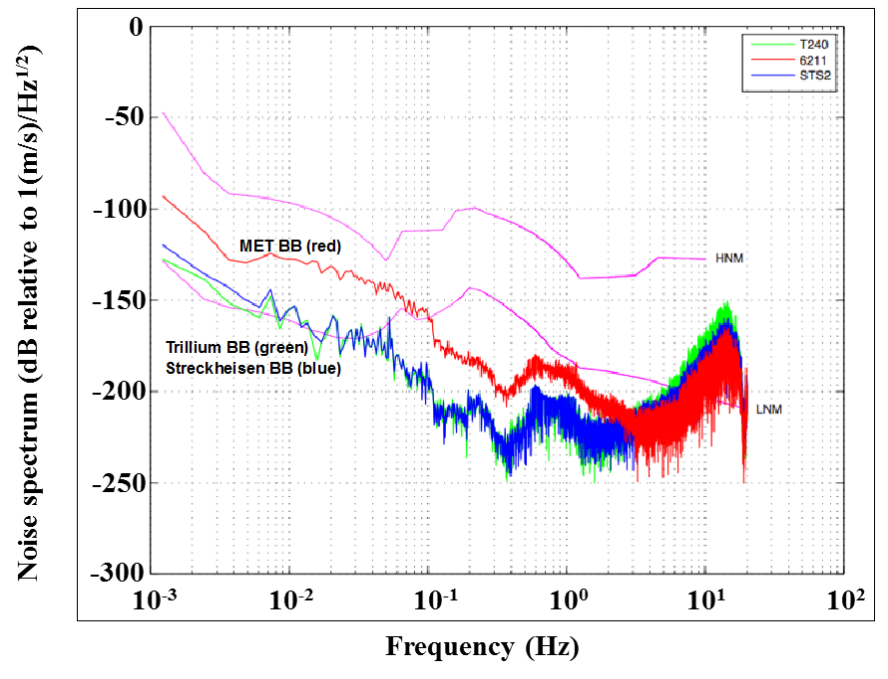
Noise performance of a MET seismometer (CME-6211) in comparison with high-performance, industry-grade broadband seismometers. Red: MET seismometer CME-6211; Blue: Streckheisen STS-2 broadband seismometer; Green: Trillium T240 broadband seismometer.

Figure 3 shows the measured noise spectrum from a high-performance MET seismometer (CME-6211) in comparison with two high-performance, industry-grade broadband seismometers, the Streckheisen STS-2 and Trillium T240 units. The experiment is conducted at the Incorporated Research Institutions for Seismology (IRIS), Program for Array Seismic Studies of the Continental Lithosphere (PASSCAL) instrument center and EarthScope USArray array operations facility. The noise performance of the MET sensor is very similar with those high-performance devices up to periods of 60 seconds, demonstrating the feasibility of MET technology for broadband seismology.

## 3. Different Types of MET Motion Sensor

**Figure 4.**
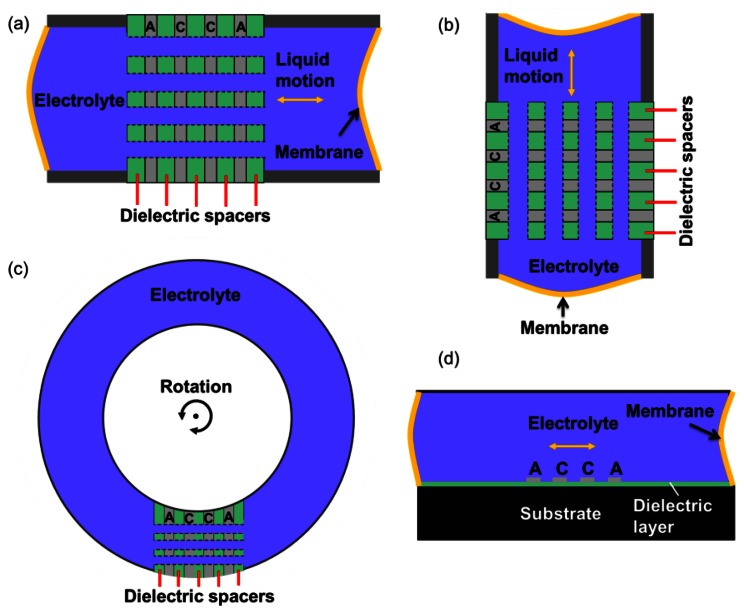
Schematic of different type of MET motion sensor.

Motion sensors based on MET technology include linear and angular accelerometers, rate sensors, gyroscopes and seismometers. Linear MET sensors can be configured for both horizontal and vertical sensing, as shown in Figure 4(a and b).

In conventional MET sensors, platinum mesh and porous dielectric are adhesively bonded to form the electrodes and insulator layers illustrated in Figure 1. In addition to the sensing element, highly-flexible rubber membranes are manually assembled at the two ends of the channel to seal the electrolyte, providing restoring force for the mechanical subsystem. Examples of the performance parameters for commercially available MET linear seismic sensors are listed in Table 1. MTSS 1001 is a single-axis, ultra-compact, high gain, 1-Hz seismic sensor. MTSS 2003 contains three orthogonal oriented MTSS 1001 seismic sensors. It is probably the world smallest tri-axis 1-Hz seismometer. CME-6211 is a high performance MET seismometer, which is mentioned above. EP-300 is a very broadband seismometer by eentec [26].

**Figure 5.**
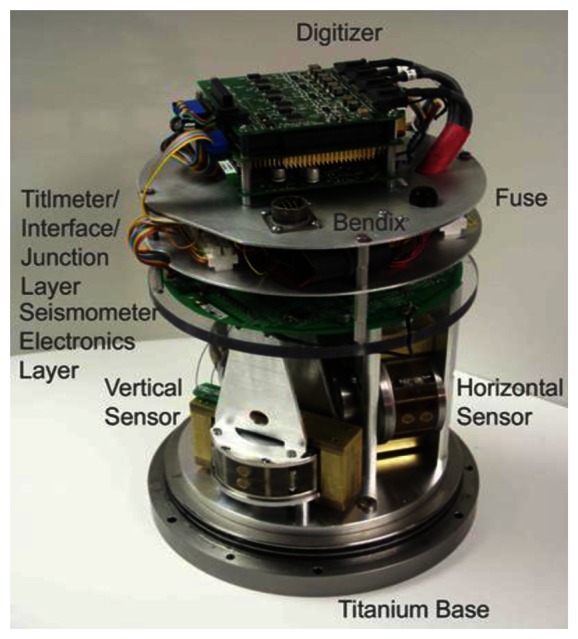
An assembled 3-axis linear MET seismometer including two orthogonal horizontal sensors and one vertical sensor, along with the sensing circuits.

Figure 5 shows an assembled 3-axis linear MET seismometer including two orthogonal horizontal sensors and one vertical sensor, along with the sensing circuits.

Figure 4(c) shows the schematic of configuration of a rotary MET sensor. If an angular acceleration is applied as shown by rounded arrow, the electrolyte flows through the sensitive element. This is another application of the technology. Currently the MET angular sensor is probably the only technology which commercially delivers highly sensitive rotational seismometers with a very low self-noise. Table 2 shows the key performances of current commercially available rotational seismometers (METR-03 and R2) [27,28].

The key advantages of MET sensors from other inertial sensors include, but are not limited to:

(1) The inertial mass is a liquid (electrolyte solution flowing through the transducer) and no moving mechanics subject to wear out and possible damage, which makes the performance more reliable and enables inherent ability to withstand high shock forces.
(2) The sensitivity of this sensor does not depend on the direction of sensitivity axis in space.
(3) High sensitivity and low self-noise at low and ultra-low frequency ranges or even DC with the liquid inertial mass, properly selected parameters of the transducer and the proper structural design.

Despite the rather high output parameters obtained, the conventional MET devices developed and produced at present have a number of faults that ultimately limit their application range. The main ones are as follows:

(1) High cost of transducer manufacturing;
(2) High scatter of parameters of manually produced transducers resulting in the necessity of individual tuning of the corresponding electronics for each sensor, which also increases the cost of the device;
(3) Early decrease in sensitivity of the sensor in the high–frequency range;
(4) Very limited possibilities to optimize the cell parameters to decrease the instrument self-noise.

**Figure 6.**
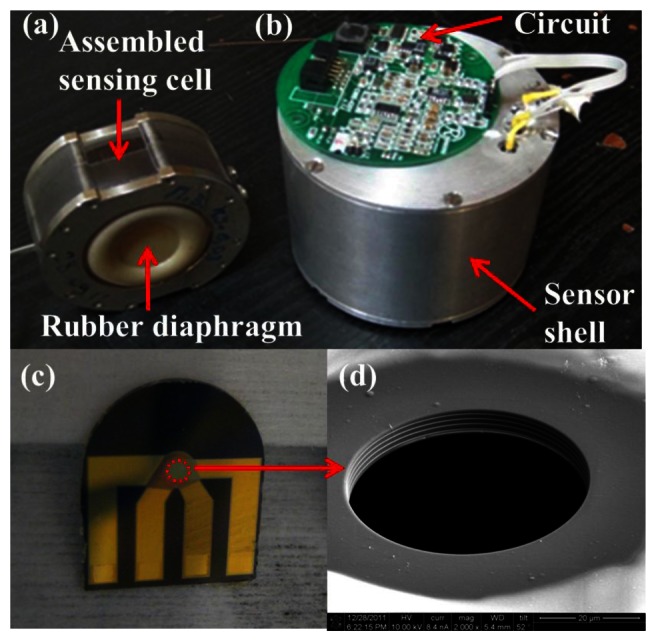
Fabricated MEMS MET seismometer. (**a**) assembled MET sensing cell, (**b**) sensor circuits and protection cell, (**c**) unpackaged MEMS MET core, and (**d**) Zoom-in view of the MET sensing element from scanning electron microscopy (SEM) showing stacked alternating platinum (electrodes) and silicon nitride (dielectric spacers).

These shortcomings can be overcome by introducing MEMS microfabrication techniques to build the MET sensing element, as shown in Figure 6 [18], which reduces the MET cell size and produced the internal dimensions close to 1 μm. The use of MEMS allows batch fabrication, thereby lower the cost, improves the sensitivity and reproducibility of the device, and has reached 1 micro Gee noise level.

**Figure 7.**
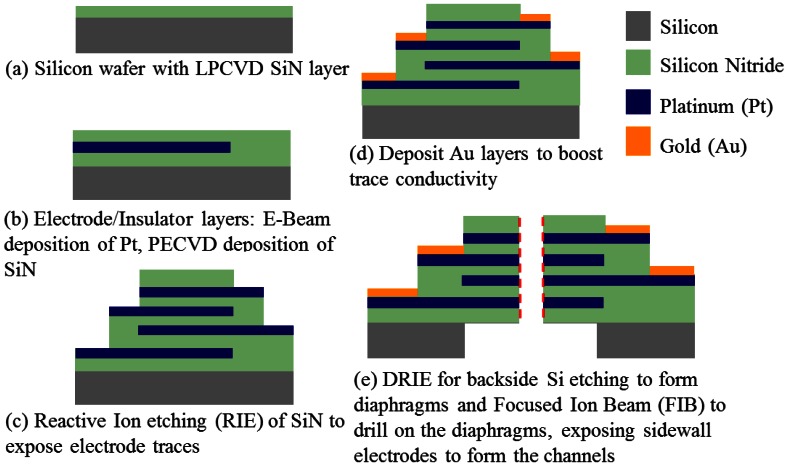
Fabrication process of the MEMS MET seismometer core.

Figure 7 details the fabrication processes. A silicon wafer with a low-pressure chemical vapor-deposited (LPCVD) silicon nitride (SiN) layer on top forms the substrate of the device. Four electrodes are 10 nm/200 nm Ti/Pt layers deposited by an E-beam evaporator and followed by lift-off. Each electrode is separated by a 1 μm plasma-enhanced chemical vapor-deposited (PECVD) SiN layer and branches to a separate contact pad used for electrical connection to the external circuitry. Finally, focused ion beam (FIB) and deep reactive ion etching (DRIE) are used to drill the holes and etch backside silicon respectively to form the channels. Optical and SEM images of the fabricated single-50 μm-diameter-channel device are shown in Figure 6(c and d).

**Figure 8.**
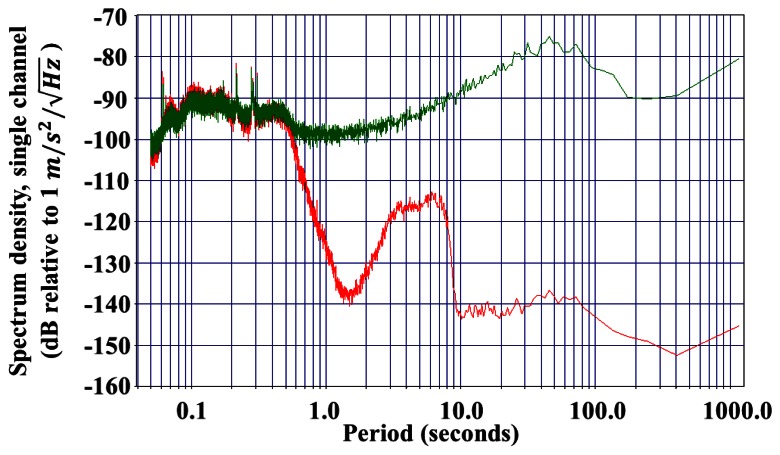
Spectrum density of a fabricated single-50 μm diameter-channel MEMS MET seismometer in comparison with a commercialized seismometer CMG-40T (Guralp Systems Ltd.). **Red**—CMG-40T, **Green**—MEMS MET seismometer.

The self-noise spectrum density achieves $1.0\times 10^{-5\;}m/s^{2}/\sqrt{Hz}$ at 1 Hz for the current device, and the sensitivity achieves 400 V/(m/s^2^) [18]. Figure 8 shows the spectrum density of a fabricated single-50 μm diameter-channel MEMS MET seismometer in comparison with a commercialized seismometer CMG-40T (Guralp Systems Ltd.).

**Figure 9.**
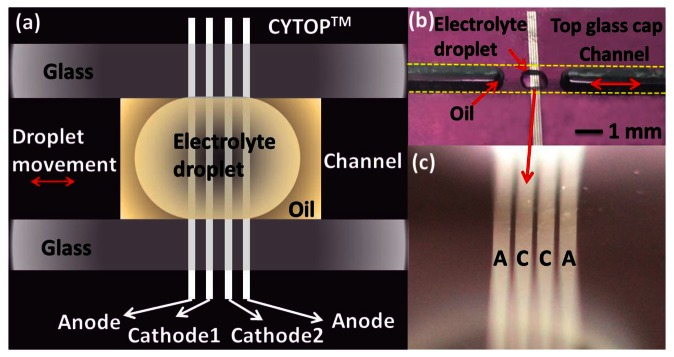
Schematic of the droplet-based MET accelerometer, (**a**) An overview, (**b**) Optical image of the accelerometer core, (**c**) Zoom-in view of the droplet-covered electrodes.

**Figure 10.**
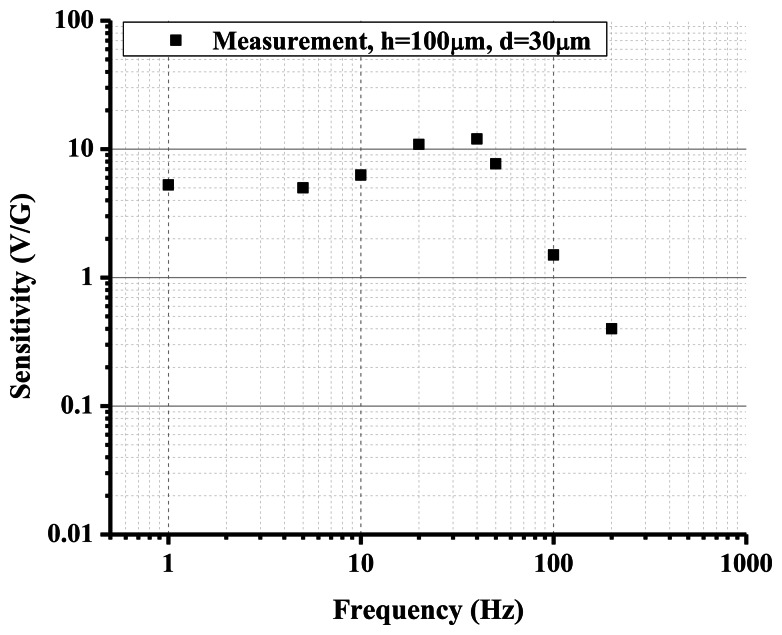
Measured sensitivity frequency response of the droplet-based micro MET accelerometer.

Figure 4(d) is an alternative sensing element configuration with planar electrodes. This structure is studied in [20] and it is shown that it has promising performance comparable with ring electrodes in open holes when the device size is reduced to micro-scale. This planar electrode structure is very easy to fabricate with standard photolithography technology, offering outstanding precision and repeatability. Furthermore, on the micro-scale, the relative flat and smooth surface will produce much less disturbance to the flow when there is electrolyte liquid motion under external acceleration than is found with the ring electrode structure. This configuration can be applied to both linear and angular MET motion sensors. An electrolyte droplet-based low frequency micro-accelerometer based on this planar electrode structure has been developed [29]. Inspired by the promising and unique performance of MET and advantages of silicon-based planar microfabrication technique with small size, low cost, outstanding precision and repeatability, the device employs a sub-microliter electrolyte droplet encapsulated in oil as the sensing body and MET electrodes as read-out mechanism. Figure 9 illustrates the device consisting of a planar four-electrode (anode-cathode-cathode-anode) MET cell located in a solid rectangular housing channel on a silicon substrate with LPCVD silicon nitride. Four electrodes made of 10 nm/100 nm Ti/Pt are deposited by E-beam evaporation and patterned with lift-off process. Then, surface modification is performed utilizing a hydrophobic thin film coating on top of the electrodes with proper patterning (hydrophilic spots surrounded by the hydrophobic areas). These hydrophilic spots anchor the droplets and the surrounding hydrophobic area acts as feedback system allowing the droplets to stabilize in the center hydrophilic spot in the case of high external acceleration input. Before final assembly of the device, a 0.8 μL concentrated iodine-iodide electrolyte droplet and a small amount of mineral oil are sequentially dispensed by micropipettes in the hydrophilic area covering platinum electrodes. The oil not only prevents the electrolyte droplet from evaporating (maintaining constant ion concentration), but also works as the elastic diaphragm to provide a restoring force. Finally, the glass housing channel is assembled. Figure 10 shows the measured sensitivity frequency response of a droplet based MET accelerometer with electrode width of h=100 μ*m*, and spacing of *d*=30 μm. The device achieves sensitivity of 10.8V/G (G=9.81 m/S^2^) at 20 Hz with nearly flat response over the frequency range of 1–40 Hz and a low noise floor of $100\;µG/\sqrt{Hz}$ at 20 Hz. Furthermore, the novel idea of using oil film as sealing diaphragm eliminates the complicated three-dimensional (3D) packaging used in both conventional and MEMS based MET sensors.

## 4. Conclusions

In conclusion, molecular electronic transducers comprising a simple set of four-electrodes, a liquid-state electrolyte as inertial mass and housing show excellent ability to be applied in motion sensors, including linear/angular accelerometers, gyroscopes and seismometers. The combination of high sensitivity, large dynamic range, wide pass band, and low self-noise distinguishes MET sensors from conventional MEMS motion sensors. The unique principles behind MET sensors also contribute to small size, simple and low-cost fabrication, low power consumption, high shock sustainability and independence of installation angle. Deployment of MEMS microfabrication in building MET core sensing element, resulting in 1 μm internal dimension, improves the sensitivity and reproducibility of the device. The performance of MET sensors can be easily optimized for a variety of applications by adjusting the geometry or configuration of the sensing element, especially implementing MEMS. The mechanism of MET provides a new paradigm of next generation high performance liquid-state motion sensor.

## Figures and Tables

**Table 1.** Current performance parameters for linear MET seismic sensors.

**Performance**	**MTSS 1001**	**MTSS 2003**	**EP-300**	**CME-6211**
Frequency range	1–300 Hz	1–300 Hz	0.0167–50 Hz	0.0167–50 Hz
Sensor Noise	$50\;\text{ng}/\sqrt{Hz}$	$50\;\text{ng}/\sqrt{Hz}$	$10\;\text{ng}/\sqrt{Hz}$	$10\;\text{ng}/\sqrt{Hz}$
Conversion Factor	300 V/(m/s)	300 V/(m/s)	2,000 V/(m/s)	2,000 V/(m/s)
Dynamic Range	>110 dB	>110 dB	150 dB at 1 Hz	135 dB
Non-linearity	<0.2%	<0.2%	Not specified	<1%
Orientation	Any direction	Any direction		Any direction
Weight	180 g	250 g	9.5 kg	7.5 kg
Temperature	“−40−+65 °C”	“−40 − +65 °C”	−12 − +55 °C”	−40 − +75 °C”
Power	<80 mW	<250 mW	<250 mW	<250 mW

**Table 2.** Current performance parameters for MET rotational seismometers.

**Performance**	**METR-03**	**R2**
Output	angular rate	angular rate
Noise at 1 Hz	$8\times 10^{-7}\text{rad}/s^{2}/\sqrt{Hz}$	$5\times 10^{-7}\text{rad}/s^{2}/\sqrt{Hz}$
Full Scale Range	0.1 *rad*/*s*	0.3 *rad*/*s*
Bandwidth	0.033-100 Hz	0.033 -50 Hz
Operating Temperature range	−40 − +75 °C	−40 − +75 °C
